# Supplementary material for: The interactome of intact mitochondria by cross-linking mass spectrometry provides evidence for coexisting respiratory supercomplexes
Source: Mol Cell Proteomics. 2017 Dec 8;17(2):216–32. doi: 10.1074/mcp.RA117.000470 (PMC5795388; doi:10.1074/mcp.RA117.000470)
Supplement: Supplemental Data [file supp_RA117.000470_133922_0_supp_23978_fzffwf.zip › spectra_annotation/mito_spectra_annotation/308-1-10-1-8-1.pdf]

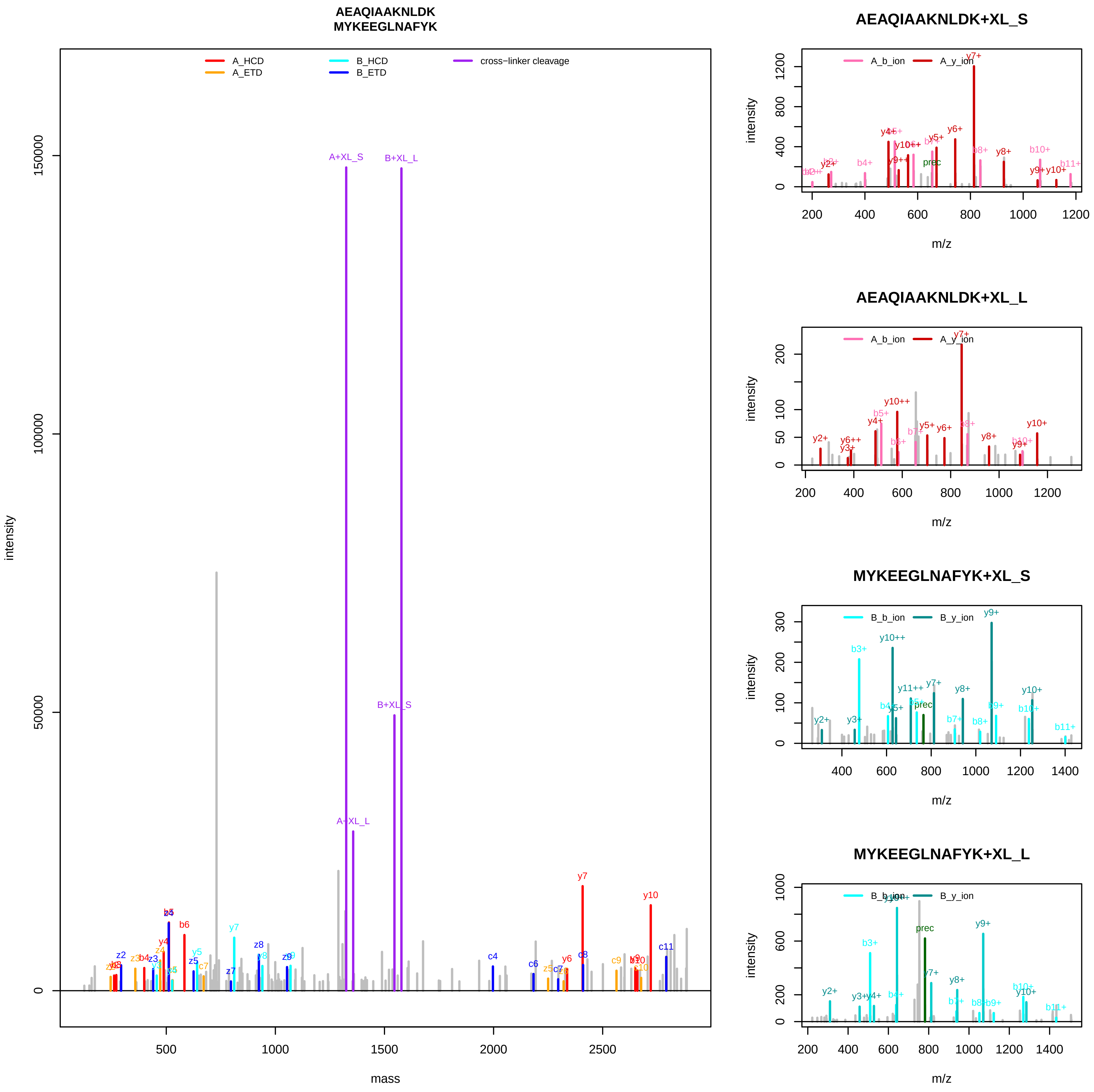
AEAQIAAKNLDK
MYKEEGLNAFYK
A_HCD
A_ETD
B_HCD
B_ETD
cross−linker cleavage
A+XL_S
B+XL_L
B+XL_S
A+XL_L
y7
y10
c11
c8
y6
c4
c6
c7
c9
z7
z8
z9
y8
z5
c7
y5
b6
b4
z2
z3
z4
y4
intensity
mass
0
50000
100000
150000
500
1000
1500
2000
2500
AEAQIAAKNLDK+XL_S
A_b_ion
A_y_ion
y7+
y6+
y5+
y4+
y8+
b10+
b11+
b8+
b4+
y2+
y9+
y10+
prec
intensity
m/z
0
400
800
1200
200
400
600
800
1000
1200
AEAQIAAKNLDK+XL_L
A_b_ion
A_y_ion
y7+
y10++
y4+
b5+
y5+
y6+
y8+
y10+
y9+
b10+
y2+
y6++
y3+
b6+
b7+
b8+
intensity
m/z
0
50
100
200
200
400
600
800
1000
1200
MYKEEGLNAFYK+XL_S
B_b_ion
B_y_ion
y9+
y10++
b3+
y11++
y7+
y8+
y10+
b10+
b11+
b9+
b8+
b7+
b4+
y5+
b5+
prec
y2+
y3+
intensity
m/z
0
100
200
300
400
600
800
1000
1200
1400
MYKEEGLNAFYK+XL_L
B_b_ion
B_y_ion
prec
y9+
b3+
y7+
y8+
b10+
y10+
b11+
b8+
b9+
b7+
y2+
y3+
y4+
b4+
intensity
m/z
0
200
600
1000
200
400
600
800
1000
1200
1400
